# Supplementary material for: The Roles of eIF4G2 in Leaky Scanning and Reinitiation on the Human Dual-Coding POLG mRNA
Source: Int J Mol Sci. 2023 Dec 5;24(24):17149. doi: 10.3390/ijms242417149 (PMC10742948; doi:10.3390/ijms242417149)
Supplement: Supplementary file 1 [file ijms-24-17149-s001.zip › Supplementary figures.pdf]

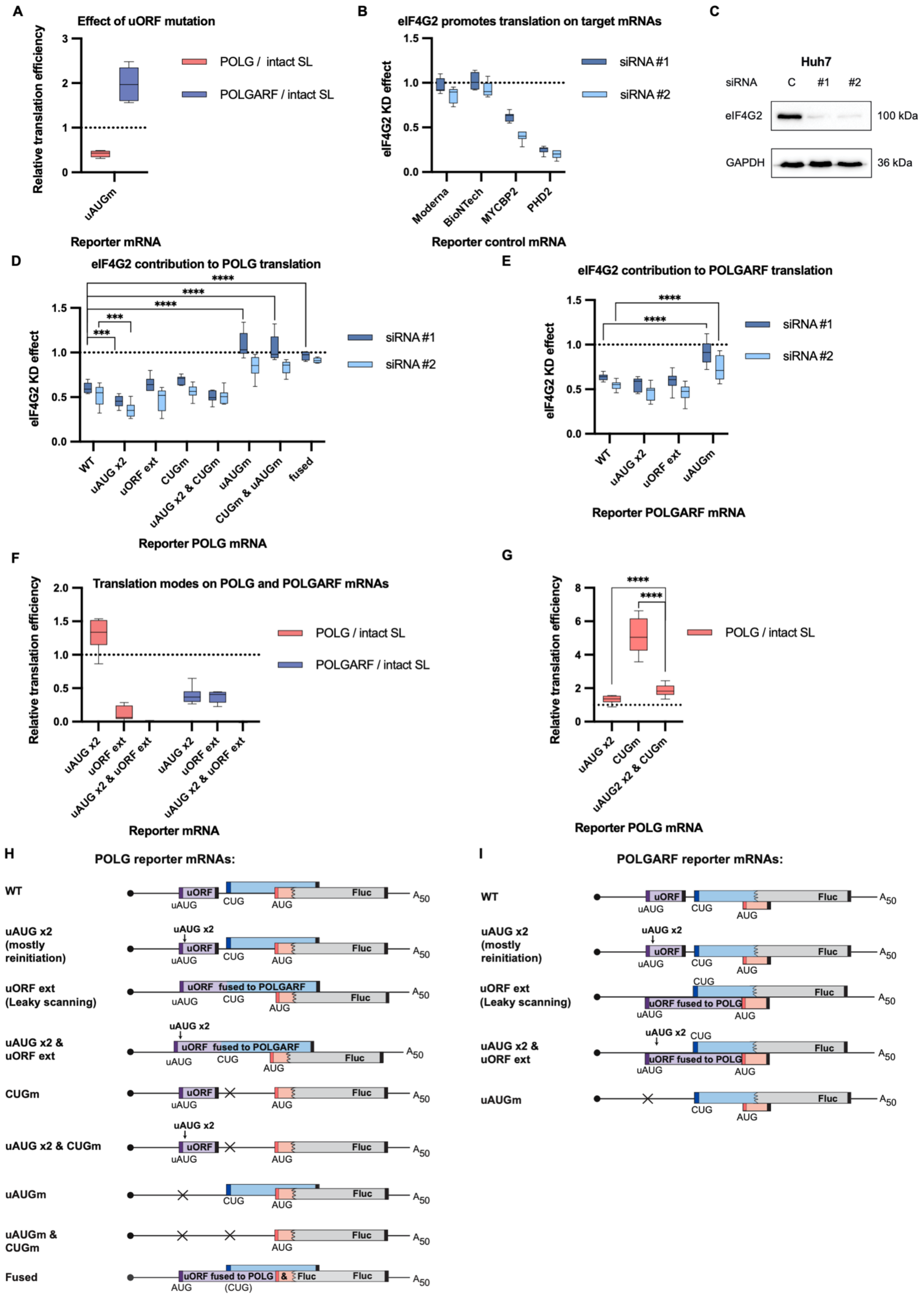
A
Effect of uORF mutation
Relative translation efficiency
POLG / intact SL
POLGARF / intact SL
uAUGm
Reporter mRNA
B
eIF4G2 promotes translation on target mRNAs
eIF4G2 KD effect
siRNA #1
siRNA #2
Moderna
BioNTech
MYCBP2
PHD2
Reporter control mRNA
C
Huh7
siRNA
C
#1
#2
eIF4G2
100 kDa
GAPDH
36 kDa
D
eIF4G2 contribution to POLG translation
eIF4G2 KD effect
****
***
siRNA #1
siRNA #2
WT
uAUG x2
uORF ext
CUGm
uAUG x2 & CUGm
uAUGm
CUGm & uAUGm
fused
Reporter POLG mRNA
E
eIF4G2 contribution to POLGARF translation
eIF4G2 KD effect
****
siRNA #1
siRNA #2
WT
uAUG x2
uORF ext
uAUGm
Reporter POLGARF mRNA
F
Translation modes on POLG and POLGARF mRNAs
Relative translation efficiency
POLG / intact SL
POLGARF / intact SL
uAUG x2
uORF ext
uAUG x2 & uORF ext
Reporter mRNA
G
Relative translation efficiency
****
POLG / intact SL
uAUG x2
CUGm
uAUG2 x2 & CUGm
Reporter POLG mRNA
H
POLG reporter mRNAs:
WT
uAUG x2 (mostly reinitiation)
uORF ext (Leaky scanning)
uAUG x2 & uORF ext
CUGm
uAUG x2 & CUGm
uAUGm
uAUGm & CUGm
Fused
uORF
uAUG
CUG
AUG
Fluc
uORF fused to POLGARF
uORF fused to POLG
&
(CUG)
A_{50}
I
POLGARF reporter mRNAs:
WT
uAUG x2 (mostly reinitiation)
uORF ext (Leaky scanning)
uAUG x2 & uORF ext
uAUGm

Supplementary Figure 1. eIF4G2 promotes POLG and POLGARF translation in Huh7 cells. The contribution of eIF4G2 to translation was assessed using reporter mRNA transfection in Huh7cells depleted of eIF4G2. Cells pretreated with either control or anti-eIF4G2 siRNAs for 72h were transfected with in vitro transcribed m^7G-capped and polyadenylated reporters. The Nluc-coding reference β-globin reporter mRNA was co-transfected with all reporters and Nluc activity was used to normalize Fluc reporter expression.

(A) Mutation of the uAUG codon affect POLG and POLGARF translation efficiency. Reporter Fluc and Nluc activies were measured in Huh7 cells treated with control siRNA. Relative translation efficiency is calculated by dividing the normalized expression of a mutant reporter by the normalized expression of a wild-type reporter ($n \geq 10$).

(B) Validation of the eIF4G2 translational targets. The data are presented as ratios of normalized reporter expression in Huh7 cells with eIF4G2 knocked down to control cells. The knockdown effect (eIF4G2 KD effect) is calculated by dividing normalized reporter expression in eIF4G2-depleted cells by normalized expression in control cells. The knockdown effect < 1 corresponds to translation inhibition in the absence of eIF4G2. Translation of reporter mRNAs with Moderna and BioNTech 5'UTRs is demonstrated as a negative control ($n \geq 6$).

(C) Western blot analysis of the eIF4G2 knockdown in Huh7cells, GAPDH as a loading control.

(D) POLG reporter mRNAs with indicated mutations were transfected into Huh7 treated either with control or anti-eIF4G2 siRNA. All assayed reporters bear intact stem-loop. The knockdown effect (eIF4G2 KD effect) is calculated by dividing normalized reporter expression in eIF4G2-depleted cells by that in control cells. The knockdown effect < 1 reflects translation inhibition by the eIF4G2 depletion. The statistical significance is determined by the Mann-Whitney U test. Three and four asterisks correspond to $p < 0.001$ and $p < 0.0001$, respectively. Statistical significancy for results obtained using siRNA #2 is omitted for the sake of readability ($p < 0.0001$ for "uAUGm", "CUGm & uAUGm", "fused" in comparison to the WT reporter).

(E) Results of POLGARF reporter mRNA transfections (similar to panel D). The stem-loop remained intact in all of assayed reporters.

(F) Contribution of leaky scanning and reinitiation to translation of POLG and POLGARF mRNAs. Relative translation efficiency is calculated by dividing the normalized expression of a mutant reporter (designated as "uAUG x2" or "uORF ext" to address leaky scanning and reinitiation, respectively) by the normalized expression of a wild-type reporter. The dotted line at 1 corresponds to translation efficiency identical to that of the wild-type reporter mRNA. Note that the contribution of reinitiation mode can be estimated by subtraction of leaky scanning mode from 1. The "uAUG x2" reporter constructs arguably reflect the upper estimate for the contribution of reinitiation mode on the wild-type mRNA.

(G) Reinitiating ribosomes mainly bypass the POLGARF CUG start codon. Relative translation efficiency is calculated in the same way as in Panel F. Statistical significance is determined by the Mann-Whitney U test. Asterisks denote $p < 0.0001$.

(H) Schematic representation of the POLG reporters examined in panel D and F (not in scale). The POLG start codon (red bar) drives translation of the chimeric reporter protein, which consists of 15 N-terminal POLG amino acids (shown in pink) fused to the firefly luciferase (Fluc). The POLGARF CUG codon (the dark blue bar) drives translation of an ORF that overlaps out-of-frame with the Fluc and encodes the 60 aa-long peptide with 33 N-terminal POLGARF amino acids (CUG-driven uORF is displayed in blue). The purple box depicts the regulatory uORF. The start codons are shown in corresponding color bars and black bars depict stop codons. The stem-loop is omitted for the sake of readability. Crosses display the positions of the upstream (with respect to the POLG ORF) start codons that were substituted for the stop codons in the corresponding reporters. In the reporter mRNA called "fused", the uORF is fused in-frame to the chimeric POLG/Fluc sequence. This resulting chimaera evaluates the role of eIF4G2 in the POLG/POLGARF mRNA translation initiation from the 5'-end to the uAUG. (D) Panel D is similar to panel C, with the exception that POLGARF reporter mRNAs are displayed. The POLGARF CUG start codon drives translation of a chimeric reporter protein, consisting of 33 N-terminal POLGARF amino acids (shown in blue) fused to the Fluc. The POLG start codon is out-of-frame with Fluc and provides translation of a 20 amino acid long stub (shown in pink).

Ribosomes can reach the main POLG start codon via leaky scanning through the uAUG or by reinitiation after completing the translation of the uORF. To estimate the eIF4G2 contribution to the leaky scanning, the uORF stop codon was mutated so that the extended uORF became fused to POLGARF ORF and overlapped out-of-frame with the firefly luciferase ORF (referred to as "uORF ext"). To significantly reduce the leaky scanning through the uORF, two extra start AUG codons (referred to as "uAUG x2") were inserted in-frame and near the uAUG codon. On such 5' UTR, ribosomes mostly reach the main POLG start codon via reinitiation. Both mutations were introduced into the POLG 5' UTR (referred to as "uAUG x2 & uORF ext") to estimate the scanning complexes retention at the uORF in-frame start codons. The reinitiated scanning complexes on their way to the POLG start codon might interfere with the 80S ribosomes on the POLGARF uORF, increasing the need for eIF4G2. To estimate if this is the case, we compare the eIF4G2 contribution to reinitiation on the "POLG uAUG x2" reporter with that on corresponding

reporter mRNA with mutated POLGARF start codon (referred to as "uAUG x2 & CUGm"). The start codons are shown in corresponding color bars and black bars depict stop codons.

(I) Panel I is similar to panel H, with the exception that the POLGARF reporter mRNAs are displayed. The POLGARF CUG start codon drives translation of a chimeric reporter protein, consisting of 33 N-terminal POLGARF amino acids (shown in blue) fused to the Fluc. The POLG start codon is out-of-frame with Fluc and provides translation of a 20 amino acid long stub (shown in pink). Cross indicates substitution of the stop codon for the uAUG. The uORF stop codon was mutated so that the extended uORF became fused to the POLG ORF stub and overlapped out-of-frame with the firefly luciferase ORF (referred to as “uORF ext”). The POLGARF “uAUG x2” and “uAUG x2 & uORF ext” reporters are constructed similarly to corresponding POLG reporters.

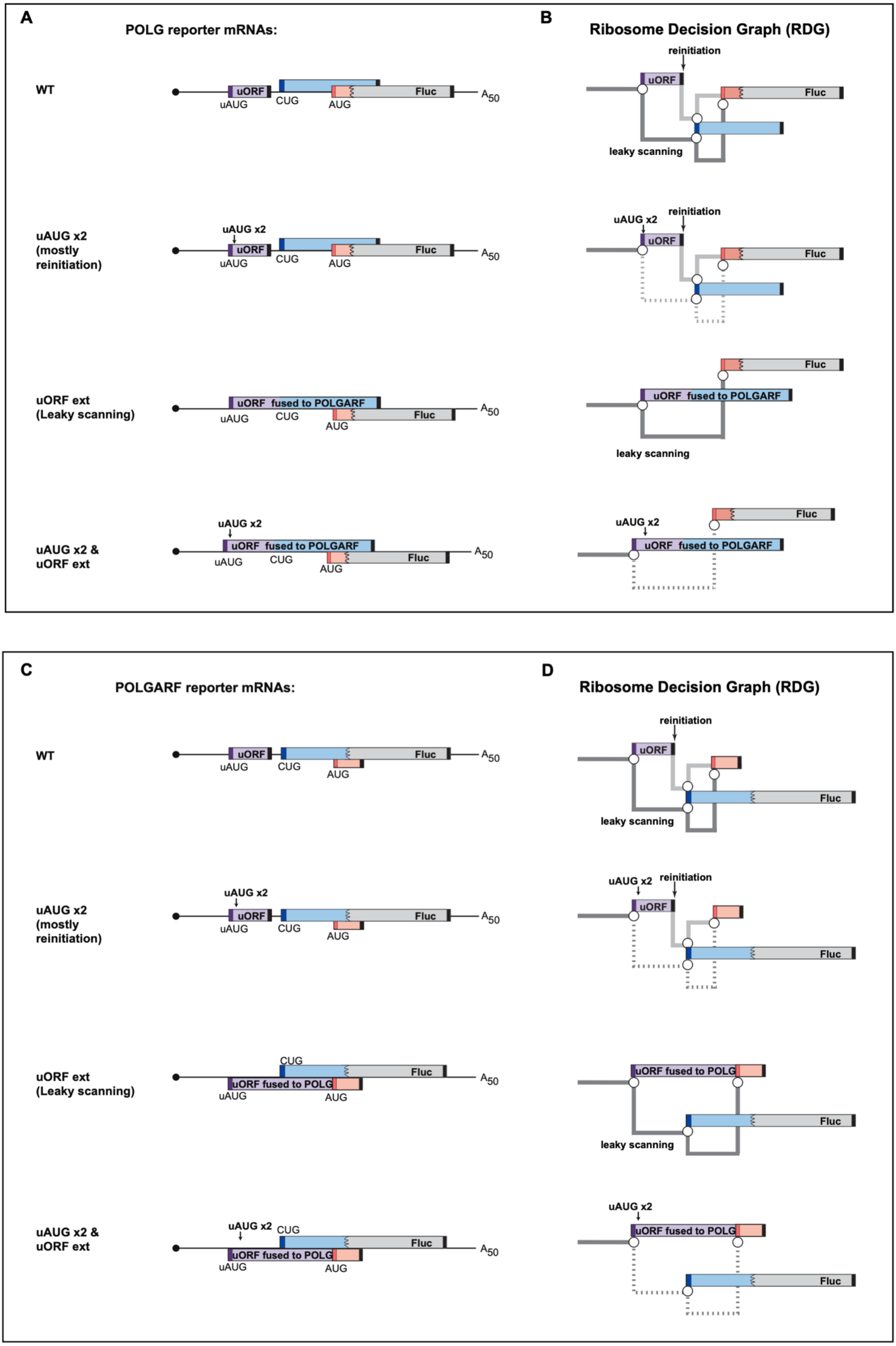
A
POLG reporter mRNAs:
WT
uORF
uAUG
CUG
AUG
Fluc
A50
uAUG x2
(mostly
reinitiation)
uORF ext
(Leaky scanning)
uORF fused to POLGARF
uAUG x2 &
uORF ext
B
Ribosome Decision Graph (RDG)
reinitiation
leaky scanning
C
POLGARF reporter mRNAs:
uORF fused to POLG
D
Ribosome Decision Graph (RDG)

Supplementary Figure 2. Ribosome paths to initiate POLG and POLGARF translation. Please note that the scheme has not been drawn to scale for readability.

(A) Schematic representation of the POLG reporters used to study eIF4G2 contribution to leaky scanning and reinitiation on POLG mRNA (not in scale). The POLG start codon (red arc) drives translation of the chimeric reporter protein, which consists of 15 N-terminal POLG amino acids (shown in pink) fused to the firefly luciferase (Fluc). The POLGARF CUG codon (dark blue arc) is out-of-frame with Fluc and drives translation of an uORF that overlaps with Fluc sequence and encodes 33 N-terminal POLGARF amino acids fused to some amino acids de-rived from Fluc sequence (60-aa peptide in total, CUG-driven uORF is displayed in blue). The purple box depicts the regulatory uORF. The start codons are shown in corresponding color arcs and black arcs denote stop codons. The stem-loop is not shown for the sake of readability. Ribosomes can reach the main POLG start codon via leaky scanning through the uAUG or by reinitiation after completing the translation of the uORF. To estimate the eIF4G2 contribution to the leaky scanning, the uORF stop codon was mutated so that the extended uORF became fused to POLGARF ORF and overlapped out-of-frame with the firefly luciferase ORF (referred to as “uORF ext”). To significantly reduce the leaky scanning through the uORF, two extra start AUG codons (referred to as “uAUG x2”) were inserted in-frame and near the uAUG codon. On such 5' UTR, ribosomes mostly reach the main POLG start codon via reinitiation. Both mutations were introduced into the POLG 5' UTR (referred to as “uAUG x2 & uORF ext”) to estimate the scanning complexes retention at the uORF in-frame start codons.

B) Ribosome Decision Graphs representing translation as multiple ribosome paths through the wild-type or mutated POLG 5′ UTRs. Boxes demonstrate ORFs. Circles denote branching points where the ribosome makes a “decision” of whether to initiate or not. The path of leaky scanning complexes towards the downstream start codons is shown in dark grey. Light grey path represents the post-terminating small ribosome subunit that resumes scanning and can initiate on the downstream POLGARF or POLG start codons (reinitiation path). The visualization concept was adopted from [26]. The dotted paths represent the significant decrease in the corresponding mode of initiation.

(C) Schematic representation of the POLG reporters used to study eIF4G2 contribution to leaky scanning and reinitiation to POLGARF translation (not in scale). The POLGARF CUG start codon drives translation of a chimeric reporter protein, consisting of 33 N-terminal POLGARF amino acids (shown in blue) fused to the Fluc. The POLG start codon is out-of-frame with Fluc and provides translation of a 20 amino acid long stub (shown in pink). The uORF stop codon was mutated so that the extended uORF became fused to the POLG ORF stub and overlapped out-of-frame with the firefly luciferase ORF (referred to as “uORF ext”). The POLGARF “uAUG x2” and “uAUG x2 & uORF ext” reporters are constructed similarly to corresponding POLG reporters.

(D) Panel D is similar to panel B. Ribosome Decision Graphs representing translation as multiple ribosome paths through the wild-type or mutated POLGARF 5′ UTRs.

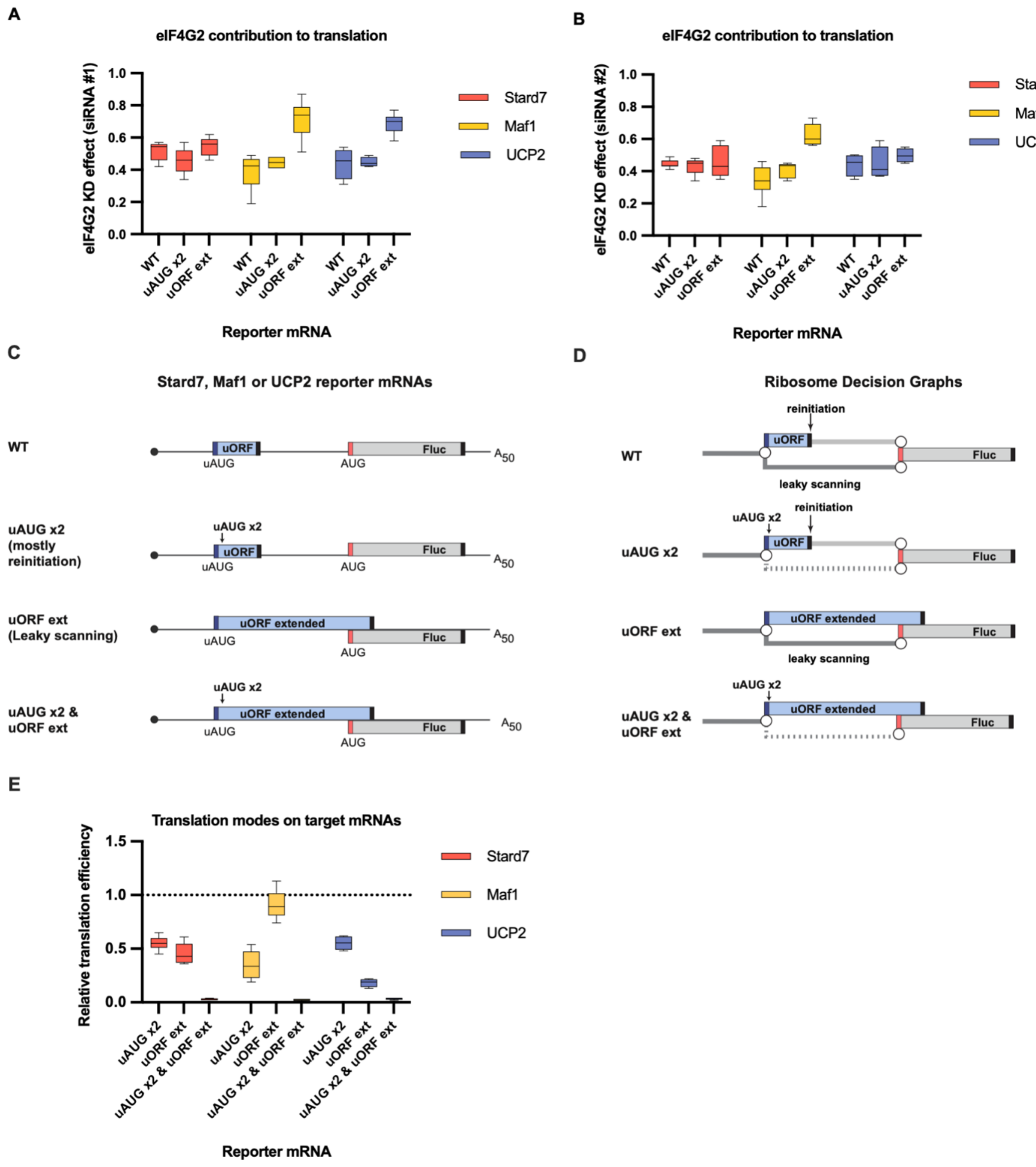

Supplementary Figure 3. eIF4G2 promotes both leaky scanning and reinitiation on Stard7, Maf1, and UCP2 mRNAs in Huh7 cells. (A) *In vitro* transcribed m^{7}G-capped and polyadenylated Maf1, Stard7, and UCP2 reporters with the indicated wild-type (WT) or mutated 5' UTRs were transfected into mock- and eIF4G2-depleted (siRNA#1) Huh7 cells alongside with reference β-globin reporter mRNA ($n \geq 10$). The effect of the knockdown (eIF4G2 KD effect) is calculated by dividing normalized reporter expression in eIF4G2-depleted cells by normalized expression in control cells. The knockdown effect < 1 shows translation inhibition in the absence of eIF4G2.

(B) Panels B is similar to panel A, except siRNA #2 was used for the eIF4G2 depletion in Huh7 cells ($n \geq 10$).

(C) Schematic representation of the reporters examined in panels A, B, and E (not in scale). Stard7, Maf1, and UCP2 5'UTRs contain uORFs (shown in blue box). Dark blue and red bars depict uAUG and main start codons respectively, black bars depict stop codons. All analyzed 5' UTRs were mutated to address leaky scanning and reinitiation separately. Leaky scanning is addressed using an mRNA reporter with an extended uORF that overlaps significantly with the Fluc coding sequence (referred to as "uORF ext"). The insertion of two additional uAUGs into uORF (designated as "uAUG x2") makes the reinitiation nearly the only way for ribosomes to reach the main start codon. To estimate the scanning complexes retention at the uORF in-frame start codons, both mutations were introduced into these 5' UTRs (referred to as "uAUG x2 & uORF ext").

(D) Ribosome Decision Graphs representing translation as multiple ribosome paths through the wild-type or mutated 5′ UTRs. Boxes demonstrate ORFs. Circles depict branching points where the ribosome makes a “decision” of whether to initiate or not. The path of leaky scanning complexes towards the downstream start codon is shown in dark grey. Light grey paths represent the post-terminating small ribosome subunit that resumes scanning and can initiate on the main start codon (reinitiation path). The dotted paths represent the significant decrease in the corresponding mode of initiation.

(E) The contributions of leaky scanning and reinitiation to the translation of Stard7, Maf1, and UCP2 mRNAs. Relative translation efficiency is calculated by dividing the normalized expression of a mutant reporter (designated as "uAUG x2" or "uORF ext" to address leaky scanning and reinitiation, respectively) by the normalized expression of a wild-type reporter ($n \geq 10$). The dotted line at 1 corresponds to translation efficiency identical to that of the wild-type reporter mRNA. Note that contribution of reinitiation mode can be estimated by subtraction of leaky scanning mode from 1. The “uAUG x2” reporter constructs reflect the upper estimate for contribution of reinitiation mode on the wild-type mRNA.
